# Breeding consequences of flavivirus infection in the collared flycatcher

**DOI:** 10.1186/s12862-018-1121-5

**Published:** 2018-02-05

**Authors:** Tanja M. Strand, Åke Lundkvist, Björn Olsen, Lars Gustafsson

**Affiliations:** 1 Department of Medical Biochemistry and Microbiology, Zoonosis Science Center, Uppsala University, Box 582, SE-751 23 Uppsala, Sweden; 2 Department of Medical Sciences, Uppsala, Sweden; 3 Department of Animal Ecology/ Ecology and Genetics, Uppsala University, Uppsala, Sweden; 4 Present Address: National Veterinary Institute (SVA), SE-75189 Uppsala, Sweden

**Keywords:** West Nile virus, Flavivirus, Humoral immune response, Host reproduction, Host ornament, Trade-off

## Abstract

**Background:**

The breeding consequences of virus infections have rarely been studied in avian natural breeding populations. In this paper we investigated the links between humoral immunity following a natural flavivirus infection and reproduction in a wild bird population of collared flycatcher (*Ficedula albicollis*). We analyzed plasma from 744 birds for antibodies and correlated these results to a number of reproductive components.

**Results:**

Nearly one third (27.8%) of the sampled collared flycatchers were found seropositive for flavivirus. Males had significantly more frequently flavivirus antibodies (32.3%) than females (25.1%). Seropositive females differed significantly from seronegative females in four traits: they had earlier lay date, higher body weight, higher survival rate and were older than seronegative females. The females did not differ in clutch size, number of fledged young or number of recruited young. Seropositive males had female partners with earlier lay date, i.e. the males bred earlier and they also produced more fledged young than seronegative males. In contrast, the males did not differ in clutch size, number of recruited young, male weight, age or survival. Interestingly, seropositive males had larger ornament, forehead badge size, than seronegative males.

**Conclusions:**

Collared flycatchers with an antibody response against flavivirus were more successful than birds with no antibody response, for any of the measured life history traits. The positive link between flavivirus antibody presence and life-history trait levels suggest that it is condition dependent in the collared flycatcher.

## Background

*Flaviviruses* is a genus containing both mosquito- and tick-borne viruses, many of them causing serious diseases in humans, such as Dengue virus infections with estimated 100–200 million human cases each year [1]. West Nile virus (WNV) is another wide-spread flavivirus [2] that is re-emerging in Europe [3]. WNV, as an example, is transmitted by ornitophilic mosquitoes between birds, where many bird species act as amplifying hosts. Sometimes, the virus is transmitted by other mosquito species to humans and horses who cannot transmit the virus further [2]. WNV is not only hosted and amplified by hundreds of species of birds, but is also pathogenic and has been demonstrated to cause detrimental effects on survival in many wild bird populations across various taxonomic groups [4–7]. Wild birds are more severely affected by WNV in the New World than in the Old World and this has been explained by the longer history of the virus in the Old World [8].

To understand and consequently prevent emerging flaviviruses it is vital to study the ecological details of the transmission cycles. What are the breeding consequences for birds that have survived a WNV infection? Can a flavivirus infection affect host’s life history in terms of traits such as egg laying date, clutch sizes, reproductive success and adult survival? Very little is known on how a flavivirus infection affects the reproductive success of bird populations, and no converging conclusion can be provided summarizing the few existing reports [9–13].

Our current work reported here is a rare observational study of flavivirus infection and reproductive success in wild birds. We investigated if wild birds with or without antibodies against flavivirus have varying breeding success and explored in detail how natural flavivirus infection affects flycatchers host fitness. Our study species is the collared flycatcher (*Ficedula albicollis*), a small long-distance migrant passerine belonging to the Old World flycatchers (*Muscicapidae*) [14] that has become one of the most well-studied avian species in evolutionary and ecological research [15–19]. The study population of the collared flycatcher winter in an area in which different flaviviruses including WNV are endemic [20–22].

## Methods

### Sampling population and fitness measures

Collared flycatchers are migratory, hole-nesting small passerines. We sampled a well-studied nest box breeding population of collared flycatchers on the Swedish island of Gotland in the Baltic Sea (57°10′ N, 18°20′ E). This population has been studied since 1980, in at present about 3000 nest boxes and 1000 breeding pairs. Given high site fidelity and limited dispersal [23], it is possible to keep detailed records of individuals, their life-histories throughout their lifetime. All adult birds in this study have fulfilled at least one long round-trip migration to their wintering area in Africa (southern Congo, Angola, Zambia and Botswana, data from our own study using geolocators [20]). We sampled the birds in the feeding period of offspring, June 2011. All nest boxes were visited regularly to determine date of egg laying (every fourth day), clutch size (once during incubation), hatching date of eggs (every day around predicted day), and number of fledged offspring (once after fledging). Females were caught mainly in the nest box when incubating, and males when they were feeding the young. Adults were weighed and age was determined from original year of ringing. A recruit is an offspring that has survived the following winter and returned to the breeding grounds the next year. Survival of adults to next or subsequent years was also measured. None of the individuals showed clinical signs of disease at the time of sampling. Males of this species have a condition-dependent white patch of feathers on the forehead that has been demonstrated to be a secondary sexual trait [16, 24]. The area of this white forehead patch was determined from the length and width measured with calipers to the nearest 0.1 mm on live birds after catching.

### Sample collection

Blood was collected from the brachial vein in the wing from nearly 750 wild-caught adult birds using sterile syringes, into EDTA-microfil tubes. Plasma was separated from the cellular part by centrifugation of the blood samples. Resulting plasma was stored at − 70°C. Additionally, faecal samples were collected for screening of an active virus excretion. Flaviviruses have been detected in birds from cloacae swab samples in previous studies [25, 26]. Faecal samples were taken from 300 birds, and placed in virus transport medium (Hanks balanced salt solution containing 0.5% lactalbumin, 10% glycerol, 200 U/ml penicillin, 200 μg/ml streptomycin, 100 U/ml polymyxin B sulphate, and 250 μg/ml gentamycin, and 50 U/ml nystatin; Sigma) in 2011. Faecal samples were further collected from 202 birds in 2013 and placed into empty tubes and frozen immediately to − 70°C.

### Laboratory analyses

#### Serology

Our aim was to detect WNV-specific antibodies but due to pronounced cross-reactions between different flaviviruses the screening of antibodies against WNV requires a two-step process [27]. First, a general screening of samples with a flavivirus-specific blocking Enzyme-linked Immunosorbent Assay (ELISA) technique to test for antibodies against flaviviruses in general [28], followed by further testing of the subset of the samples found positive by the blocking-ELISA (≥30% inhibition) by a WNV neutralization test (NT) [28, 29], see details below.

#### Flavivirus-specific blocking-ELISA

To determine the amount of flavivirus-specific antibodies, we used a blocking-ELISA technique [28, 30]. ELISA 96-well microplates (VWR International, Stockholm, Sweden) were incubated overnight at 4 °C. Two thirds of the plate were incubated with 1:200 WNV antigen (prepared from both a lineage 1 (“WN_0304”, Israel WNV strain) and a lineage 2 (“MgAn 798”, Madagascar WNV strain) diluted in carbonate–bicarbonate buffer (0.1 M, pH = 9·6,), and one third of the plate with “negative antigen” (uninfected cell extracts, produced as control to the WNV antigen) (100 μl/well). After washing 4 times with 300 μl 0.9% NaCl containing 0.05% Tween 20 (Sigma-Aldrich, Schnelldorf, Germany) the plates were blocked with 200 μl 3% Bovine Serum Albumin (BSA) in Phosphate Buffer Saline (PBS) for 1 h at room temperature. The plates were subsequently incubated with plasma samples for 2 h at room temperature. Positive (WNV infected) control chicken sera (1:40), negative control chicken sera and test plasma was diluted 1:20 in ELISA buffer (PBS with 0.5% BSA and 0.5% Tween-20). Each plasma sample was divided into two wells of WNV antigen coated wells and one well of “negative antigen” (50 μl/well). After incubation, a flavivirus group-specific anti-E protein monoclonal antibody (Mab) 4G2 [31] produced by the hybridoma cell line HB-112 (American-Type Culture Collection; LGC Standards AB, Boras, Sweden) was added for 1 h at RT. The Mab 4G2 was prepared against Dengue 2 and has earlier been demonstrated to react with all Dengue serotypes (1–4), Banzi, Ilheus, Japanese Encephalitis, Kunjin, Langat, Ntaya, St. Louis encephalitis, West Nile, Zika virus and weakly to Yellow fever [31]. The secondary antibody (50 μl 1:5000 Peroxidase-AffiniPure Goat Anti-Mouse IgG + IgM (H + L) (Jackson ImmunoResearch Labs, Pennsylvania, USA, Cat# 115–035-068 RRID: AB_2338505) diluted in ELISA buffer) was added after washing. The last incubation of 1 h at room temperature was followed by a last washing procedure (4 times), before 50 μl of substrate (TMB) was added to each well. When a firm blue colour developed (after approx. 5 min), H_2_SO_4_ (2.5 M) was added to the plate to stop the reaction, and the plate was read by a spectrophotometer at 450 nm. The percent inhibition (PI) of MAb binding for each test sample was calculated using the formula 100-(Optical Density (OD) mean for test serum – OD for negative antigen)/ (OD mean for uninfected chicken serum-OD for negative antigen) × 100 [30]. A threshold cut-off inhibition of 30% or more was used to determine seropositive individuals [28, 32]. A subset of the seropositive individuals was run in the Neutralization Test [28, 32].

#### West Nile virus neutralization test (NT)

NT was performed in a Biosafety Level-3 laboratory. This particular NT is based on a 96-well technique [28, 29] with modifications as follows. The virus strain used was the West Nile virus strain WN_0304 (Lineage 1, accession number AF375045 [33]), and the assay was performed using confluent Vero cells grown in 96-well plates. Plasma used in the assay were inactivated in water bath at 56 °C for 30 min. Negative sera (human) were diluted four-fold (from 1:10 to 1:10,240) in medium (Minimal Essential Medium with Earl’s salt supplemented with 1% PSN (Penicillin Streptomycin Neomycin), 1% HEPES buffer, 1%, L-glutamate, 4% FCS (Fetal Calf Serum); Invitrogen) and positive serum (WNV vaccinated rabbit) were diluted two-fold in medium, both in duplicates. To test for cross-reactivity for other flaviviruses, human sera with antibodies against Tick-borne Encephalitis virus (TBE) (vaccinated), Yellow fever (vaccinated), Japanese Encephalitis Virus (JEV) (vaccinated), and Usutu (infected blood donor) were run in two-fold serial dilutions and were correctly tested as negative. Plates also included a WNV titration in ten-fold dilutions. On each plate, at least two wells were not infected by virus and were used as blanks, their mean value subtracted from all sample values. Plasma and West Nile virus dilutions in equal volumes were incubated for 1 h at room temperature. Due to the limited bird plasma available, test samples were diluted to either 1:40 or 1:80 in medium to a single well, and virus were diluted to 1:200 or 1:500, respectively (all final concentrations). After incubation, the plasma and virus mixture were incubated at 37°C for five minutes before 200 μl was added to the 96-well tissue culture plate with Vero cell monolayer. The plates were incubated for 48 h at 37 °C in a humidified 5% CO_2_ atmosphere. The cells were inspected with a microscope and condition and the status of cell death documented. Plates were emptied and washed three times with 300 μl PBS before fixated by 200 μl 80% acetone for 90 min at − 20 °C.

Besides visual inspection of monolayers documenting the cytopathology, a second type of ELISA was performed to document the inhibition. Plates were emptied and dried for 30 min before adding 250 μl blocking buffer (3% BSA in PBS) to each well to be blocked at 4 °C over night. 100 μl/well of MAB HB-112 diluted in ELISA-buffer (PBS + 0.5% BSA + 0.005% Tween-20) at 1:2000 was added to each well, and plates were incubated at 37°C for 1 h. After four washes in washing buffer (PBS with 0,05% Tween), 100 μl/well Alkaline Phosphatase-AffiniPure F’2 Fragment Donkey Anti-Mouse IgG (H + L) antibody (Jackson ImmunoResearch Labs, Pennsylvania, USA, Cat# 715–056-151 RRID: AB_2340781) at 1:1000 was added, and incubated at 37°C for 1 h. After five washes, two tablets of phosphatase substrate (Sigma-Aldrich) were dissolved in 10 mL of diethanolamid and 60 μl levamisole. 100 μl was added to each well and incubated for about 30 min and plate was read for absorbance 405 nm in a spectrophotometer.

### Flavivirus genome excretion

#### RNA extraction

Collared flycatcher faecal samples from 2011 and 2013 were pooled within years, where five samples comprised each pool, for RNA extraction. Viral RNA from the 2011 samples was isolated using a Vet Viral NA kit (NorDiag ASA, Oslo, Norway) and a Magnatrix 8000 extraction robot (Magnetic Biosolutions, Stockholm, Sweden). Six pools from 2011 were re-extracted (due to shortage of template) with Maxwell 16 Viral Total Nucleic Acid Purification Kit (Promega, Sweden) according to kit protocol, but with 100 μl input sample and lower volume of elution buffer (20 μl). Faecal samples from 2013 were mixed with 1 ml PBS, vortexed for 1–2 min, incubated on ice 2 h to dissolve, vortexed again followed by short centrifugation. The supernatant (20 μl of each sample before pooling) was extracted using the Maxwell 16 Total Viral Nucleic Acid Purification kit, as above.

#### Flavivirus real-time quantitative PCR (qRT-PCR)

RNA was assayed using a pan-flavi one-step quantitative real-time reverse transcription (qRT-PCR) assay with degenerate primers and probes targeting the non-structural gene five (NS5) [34] with a few modifications, as follows: The assay was performed using QuantiTect Virus + ROX Viral Kit (Qiagen, Hilden, Germany) on a Corbett Life Science RotorGene 6000. We utilized the degenerated primers Flavi all S (F) and Flavi all AS (R) as stated in [34] report in combination with the Locked-Nucleic Acid (LNA) modified probe [FAM] - TG **+ G** TWY ATG T **+ G**G YTN G **+ G**R GC - [35]. The reaction mix contained 400 nM of each primer, 100 nM of probe, 10 μL of 5× QuantiTect Virus Master Mix, 0.5 μL of 100× QuantiTect Virus RT Mix, 5 μl of RNA template and DEPC-treated water up to a total volume of 50 μL. The thermo profile is as follows; 50° C for 20 min, 94° C for 5 min, and a 50 cycle including 94° C 15 s and 50° C for 45 s.

Positive controls used were WNV, JE, YFV and Dengue (DV serotype 1–4) RNA, which was extracted with TRIzol reagent (Ambion by Life technologies, Carlsbad, CA, United States) according to the manufacturer’s instructions. The assay was evaluated and a standard curve was made using a tenfold dilution series of West Nile Virus. The standard curve, generated by setting the cycle threshold (CT) value to 0.152, exhibited a high linearity with R and an R^2^ value close to 1 (0.999 and 0.998, respectively) and E-value of 0.83.

#### Statistical analyses

In the flavivirus-specific blocking ELISA we used 744 individual plasma samples. Number of individuals (N) is not the same in all tests due to incomplete data for some individuals. Individuals considered infected in the past are those with ≥30% inhibition (seropositive) [28]. Individuals that are not considered having been infected have 0 < 30% inhibition (seronegative). For this, we applied Chi-Square for analyzing difference in seroprevalence between males and females. For both sexes, we first tested if seroprevalence (PI) – (seropositive versus seronegative category) – was related to adult age categories, with General Linear Model (GLM). If the age was related with seroprevalence (PI) and the response variable, it was included in the following models. Following, we tested if PI was related with lay date, clutch size, fledged young, recruited young, incubation weight, overwinter survival and forehead patch area. The statistical software we used was JMP, SAS Institute Inc., (Cary, NC, USA).

## Results

### Avian fitness parameters are affected by flavivirus antibody status

The seroprevalence of individuals with flavivirus reactive antibodies ≥30% inhibition (in blocking-ELISA) in migratory Collared flycatchers was 27.8% (207 of 744, 95% CI [24.6, 31]). A subset of these positive individuals (81/ 207) was tested for confirmation using the WNV NT. Eight of 81 (9.9%) had neutralizing antibodies against West Nile virus. Of these eight positives, five were males and three were females. There was a significant difference in seropositivity between sexes, more males (32.3%, *n* = 91/282) than females (25,1%, *n* = 116/462) had antibodies (≥30%) against flavivirus in the blocking ELISA (**χ**^**2**^_4.425_, *p* = 0.035). Given a significant difference in prevalence by sex, the following results have been separated by sex.

### Females

**Fig. 1:**
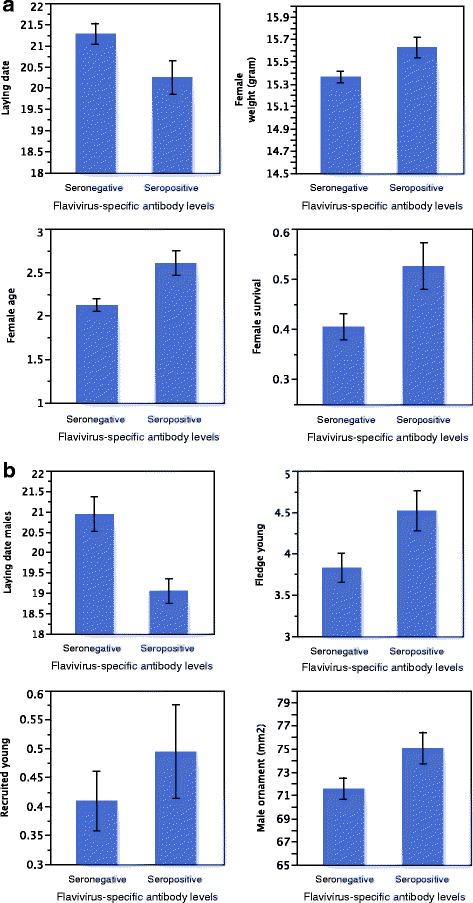
Life history traits against flavivirus specific antibody measures in the collared flycatcher. Females **a**) and males **b**) divided into two flavivirus-specific antibody levels (“Seronegative” (< 30% inhibition) and “Seropositive” (≥30% inhibition) and visualized in histograms against (mean) lay date. Females were also plotted for adult weight, adult survival (proportion annual survival) and age (in years). Males were in addition plotted for number of fledged young, number of recruited young and mean forehead patch area (ornament).

Seropositive females (≥30% inhibition) differed significantly from seronegative (< 30%) in four traits: they had earlier lay date, higher body weight, higher survival rate and were older than seronegative females (Tables 1 and 2, Fig. 1a). In contrast, they did not differ in clutch size, number of fledged young or number of recruited young (data not shown).

**Table 1:** Relation between adult age and flavivirus specific antibody measures in the collared flycatcher

	Estimate	Std Error	DF(n)	Statistical value	*P*-value
Female					
GLM Model inhibition			1(462	ChiSq = 7.68	0.0056*
Female age	−0.2066	0.0738			0.0056*
Male					
GLM Model inhibition			1(282)	ChiSq = 0.335	0.5226
Male age	0.0502	0.0871			0.5226

We tested if flavivirus specific antibody measures (negative/positive inhibition of WNV ELISA) was correlated to adult age (one year and 2 years or older), with Generalized Linear Model (GLM) bimodal model analyses

**Table 2:** Analyses of flavivirus antibody levels against the measured life history traits in the collared flycatcher

	Estimate	Std Error	DF, (n)	Statistical value	P-value
Females					
Model Lay date			2, 462	ChiSq = 14.43	0.0007
Female Age	−0.0689	0.0235		ChiSq =8.46	0.0036
Inhibition	−1.0131	0.4811		ChiSq =4.41	0.0357
Model Survival			3, 462	ChiSq = 11.64	0.0087
Lay date	0.0465	0.0220		ChiSq =4.56	0.0326
Inhibition	−0.4941	0.2215		ChiSq =4.99	0.0255
Female Age	0.1226	0.0711		ChiSq =3.04	0.0814
Model Female weight			3, 452	ChiSq = 21.99	< 0.0001
Inhibition	0.2083	0.1020		ChiSq =4.15	0.0416
Female Age	0.0979	0.0313		ChiSq =9.49	0.0021
Lay date	−0.0178	0.0099		ChiSq =3.23	0.0725
Males					
Model Lay date			2, 282	ChiSq = 14.03	0.0009
Male Age	−0.4703	0.1996		ChiSq = 5.49	0.0191
Inhibition	−1.9335	0.6336		ChiSq = 9.16	0.0025
Model Fledged Young			3, 246	ChiSq = 18.00	0.0004
Lay date	−0.0893	0.0296		ChiSq = 8.93	0.0028
Male Age	−0.1189	0.0931		ChiSq = 1.62	0.2027
Inhibition	0.7126	0.2955		ChiSq = 5.74	0.0165
Model Patch Area			1, 276	ChiSq = 4.47	0.0345
Inhibition	3.4593	1.6294		ChiSq = 4.47	0.0345

Generalized Linear Model (GLM, normal distribution) analyses of the relation between reproductive traits and flavivirus specific antibody measures (negative/positive inhibition of WNV ELISA) in male and female collared flycatcher. Relationship tested between female overwinter survival and flavivirus specific antibody response used a bimodal, maximum likelihood, Generalized Linear Model (GLM). Variables with non-significant analyses are not shown

### Males

Males with flavi-specific antibodies (≥30% inhibition) had female partners with earlier lay date, i.e. the males bred earlier, produced more fledged young and had larger ornaments (forehead patch size) than males with undetectable antibodies against flavivirus (< 30% inhibition) (Tables 1 and 2, Fig. 1b). The males did not differ in number of recruits (Fig. 1b), clutch size, male weight, age or survival (data not shown).

### Flavivirus genome excretion

The prevalence of flavivirus RNA was determined by screening faecal samples from 2011 (same year as the blood sampled birds) and 2013 using a probe based RT-PCR method. None of the tested samples (*N* = 502) were positive for flavivirus viral RNA.

## Discussion

### Flavivirus infection and general seroprevalence

Nearly one third of all collared flycatcher adults (one year or older) analyzed in this study had previously been infected by a flavivirus. Only a minority of the birds could be confirmed to have neutralizing antibodies against West Nile virus. The other flaviviruses the collared flycatchers in our study population may come across during their winter stay or migration are either well known or poorly described. These include Usutu virus [36], Tick-borne encephalitis virus [37, 38], Meaban-like virus [39], Bagaza virus (synonymous with Israel turkey meningoencephalitis virus) [40], Dengue virus, Yellow fever virus, Zika virus [22] and Louping Ill virus [41]. In Europe, West Nile virus, Usutu viruses, and Louping Ill virus cause infectious diseases in humans and have been shown to cause wild bird deaths [3, 42, 43]. We did not detect flavivirus RNA in the faecal samples of the birds, indicating that the birds did not excrete flavivirus RNA at the time of sampling. For collared flycatcher, no previous knowledge of flavivirus antibody persistence exists. For other bird species, persistence varies. For experimentally WNV-inoculated house sparrows (*Passer domesticus*), neutralizing antibody titers remain basically constant for ≥36 months for birds that survive [44]. In naturally WNV infected rock pigeons (*Columba livia*), there was no apparent antibody decay either during the experiment of 15 months [45]. In contrast, in naturally infected fish crows (*Corvus ossifragus*), WNV antibodies decayed (but still persisted) in the end of the test year [46]. We suggest that the birds with flavi-specific antibodies are the birds that have survived an acute infection, and are thus the most fit in the population with regards to both immune function against flavivirus and reproductive performance. The seronegative birds are birds that have either cleared an infection previous seasons and have seroreverted, whereby they no longer have detectable levels of antibodies, or have not come in contact with the virus.

### Sex difference

Males of this breeding collared flycatcher population were significantly more likely than females to carry flavi-specific antibodies, the opposite finding of West Nile virus studies of eastern bluebirds (*Sialia sialis*) (ELISA, females 41.6%, males 29.9%) [9], and northern cardinals (*Cardinalis cardinalis*) (ELISA and NT, females 36%, males 21%) [10]. Both eastern bluebirds and northern cardinals breed within the geographic distribution of WNV and thus the female may be at more risk of mosquito bites when incubating. In contrast, this collared flycatcher population had most likely been infected in their wintering grounds in Africa, so the infection is independent of mating or breeding behavior. Adult seropositive females are significantly older than seronegative females. We did not find an age-related pattern in males. In eastern bluebirds, age of neither females nor males (1 year old or older) was related to seroprevalence of WNV [9].

### Reproduction costs of infection

Seronegative adults were not better than seropositive adults for any of the reproductive traits, for neither females nor for males. Actually, the negative birds had the lowest seasonal values for several life-history traits in our study population. Both seropositive females and males had significantly earlier lay date than seronegative females and males. These seropositive males arrived earlier in spring in order to establish the best territories and attract (the best) females. Neither seropositive females nor males were different from seronegative females or males regarding mean clutch size, similarly reported by Marshall [10] in northern cardinals. For seropositive females there was no difference in number of fledged young compared to seronegative females. For males, the situation looked different. Seropositive males had significantly more young that fledged than seronegative males. Similar results were described in male eastern bluebirds [9], but in northern cardinals WNV seronegative females had more fledglings on average than seropositive females [10]. Speculating, the difference could be due to infection from different flaviviruses.

### Survival

There was a difference in over-winter survival for female flycatchers, where the seropositive birds had the highest survival, but not for males. The male result is similar to the results on WNV seroprevalence and survival in eastern bluebirds and northern cardinals, where no difference in survival was found [9, 10]. It is essential, however, to point out that we still do not know the potential mortality of collared flycatcher due to flavivirus infection. If many birds die due to the infection, the surviving birds with antibodies could either be of good quality, or suffer a chronic infection (however, no RNA excretion was detected).

### Sexual signaling linked to flavivirus infection

Females of many vertebrate species choose males based on secondary sexual characters, resulting in fitness consequences for a male with conspicuous ornaments. The brightness of the plumage of male greenfinches (*Carduelis chloris*) is suggested to be an ornament, and a study showed that males with brighter tail patches had higher Sindbis virus infection clearance rates and tended to produce antibodies at a higher rate than males with duller tail patches [47]. The sexual ornamental forehead patch of male collared flycatchers is condition dependent, in the sense that it is an honest signal of condition and not costly to produce [16, 48], and due to this is positively related to immune response [49]. Collared flycatchers that survive an infection can be considered of high quality and or in high condition [50, 51]. In another virus challenge study in the collared flycatcher, also in this population, the males with larger patch size produced more antibodies against Newcastle disease virus vaccine [49], which corroborates the results of this study. Specifically, flavivirus seropositive males had a significantly larger ornament patch size than seronegative males.

### Condition-dependent trade-off

This population has been utilized for two studies investigating immune response in response to Newcastle Disease virus vaccines. First, females were challenged and antibody response correlated strongly and negatively with increased reproductive effort in brood size manipulations [51]. In the second, males were challenged when establishing territory and attracting females. In this experiment, half of the males had experimentally increased breeding effort and as a result had lower levels of antibodies, demonstrating a trade-off between reproductive effort and immune response [52]. Hence, why does the flavivirus infection not lead to clear low or high reproductive success and survival in our case? One reason could be that different individuals had been infected by different flavivirus species that gives dissimilar antibody response. Another hypothesis is that these detected flaviviruses have coevolved with the birds, thereby reducing virulence and other negative effects of the infection. However, this is not the case as low response individuals have lower reproductive values. Altogether, the overall positive relationships between flavivirus antibody levels and, reproductive and survival levels in light of the concept of acquisition and allocation of resources [53], suggest condition dependence. One hypothesis emerging from a brood-size manipulation is that there is a trade-off between immune response and reproduction, resulting in a negative association between antibody levels and fitness in experimental situations relating to reproductive effort [51]. A second hypothesis, not mutually exclusive, is the “big house-big car” syndrome with high and low quality individuals [53], where some individuals can have both a strong immune defense and high reproduction, and large ornaments [49–51]. Therefore, individuals putting optimal levels of energy to immune response are the individuals with the highest fitness [54–56]. However, it should be pointed out that the seronegative group of birds consists both of individuals of high and low quality that have not been infected as well as those that possibly survived an infection and seroreverted (presumable a costly event). Whereas, the seropositive group all consists of individuals of such high quality that they survived the infection, contrary to those that might have died due to the infection before we could sample them and therefore are not included in our analyses.

Our example of breeding consequences of flavivirus infection in a wild bird demonstrates the complexity of studies in ecoimmunology, and has bearing on several topics in evolutionary ecology.

## Conclusions

We suggest that the birds with flavi-specific antibodies are the birds that have survived an acute infection, and are thus the most fit in the population with regards to immune function against flavivirus. This is supported by the fact that males in the group with flavi-specific antibody response clearly significantly had larger ornament patch size than the negative group.

Males had significantly more frequently flavivirus antibodies than females. Seronegative birds were not better than seropositive birds for any of the reproductive traits, neither for females nor for males.

Altogether, the relationships between flavivirus antibody levels and, reproductive, survival and ornament levels, in the light of the concept of acquisition and allocation of resources [53], suggest condition dependence.
